# Characterizing Tensile Strength of Building Sandstone via Uniaxial Tensile, Compressive, and Flexural Bending Tests

**DOI:** 10.3390/ma16093440

**Published:** 2023-04-28

**Authors:** Xiqiang Guan, Baofeng Huang, Zhan Li, Xiaofeng Ma, Benliang Liang

**Affiliations:** 1 College of Information, Mechanical and Electrical Engineering, Shanghai Normal University, Shanghai 201418, China; xqguan@shnu.edu.cn; 2 College of Civil Engineering, Shanghai Normal University, Shanghai 201418, China; 3 College of Civil Engineering, Nanjing Tech University, Nanjing 211800, China

**Keywords:** sandstone, tensile strength, four-point-bending strength, compression strength, stress–strain diagram, modulus of elasticity

## Abstract

Sandstone is widely used a construction and building material. However, its uniaxial tensile strength (UTS) is not adequately understood. To characterize the uniaxial tensile strength of natural sandstone, three groups of specimens were fabricated for four-point bending, uniaxial compressive, and tensile tests. To characterize the evolution of the stress–strain profiles obtained via these tests, representative expressions were developed in terms of normalized strain and strength. The magnitude of the uniaxial tensile strength exceeded that of the four-point bending strength, indicating that the uniaxial tensile strength cannot be represented by the four-point bending strength. The experimental ratio of uniaxial tensile and compression strength (33–41) was underestimated by the empirical expressions reported in the literature. The suggested correction coefficient for the FBS is 0.25. The compressive modulus (*E*_c_) was generally identical to the experimental results published in the literature, whereas the tensile modulus (*E*_t_) was overestimated. The experimental modular ratio, *E*_t_/*E*_c_, ranged from 0.12 to 0.14; it was not sensitive to Poisson’s ratio, but it increased slightly with the compressive modulus. This work can serve as a reference for computing the load-bearing capacity of sandstone components under tension.

## 1. Introduction

Natural rocks are widely used in built environments owing to their inherent prominent mechanical and physical properties and variable aesthetic effects. In the structural design of infrastructure, such as buildings, tunnels, and bridges, the mechanical properties must be known in advance. This enables the load-bearing capacity of stone components to be fully exploited without compromising the strength capacity to prevent disasters [1]. The tension strength of a rock is one of the fundamental mechanical properties in the structural design of building structures with stone columns, masonry walls, beams, and cladding [2]. Natural sandstone is commonly used in masonry walls, cladding, and other components. Regardless of the lifecycle analysis and maintenance or the structural design of new buildings with sandstone, the uniaxial tensile behavior and strength must be clearly understood. To characterize the uniaxial tensile strength (UTS) of building stones, indirect and direct tensile tests are widely used. Compared with the uniaxial compressive strength (UCS), accurately determining the direct tensile strength is more difficult. By contrast, preparing specimens for indirect tensile tests is considerably easier [3].

Indirect tensile tests, such as semicircular bending, Brazilian disc, and ring disc tests, are more convenient than direct tests in terms of specimen preparation and quality of the testing facility. Because of the aforementioned advantages, as well as their acceptable reliability, they are widely used in rock engineering. Carneiro [4] developed the Brazilian test method and it has become a standard indirect testing method to investigate the tensile strength of rock materials. Extensive analytical and experimental works have been conducted to refine the reliability of this method [5,6]. A comprehensive review by Li and Wong [7] indicated that the Brazilian test strength approximates the direct tensile strength. The size/shape effect has been proven as the main cause of the inaccuracy of experimental results [8]. Accordingly, Wang et al. [9] proposed the use of a flattened Brazilian disc to determine the UTS and other mechanical properties of rock materials.

Experimental results indicate that for most rocks, the direct tensile strength is approximately 70–90% of the Brazilian test strength [10]. Liu et al. [11] compared the experimental tensile strength of granite obtained by three methods: Brazilian tests with International Society for Rock Mechanics (ISRM) and Chinese standards [12,13] and direct tensile tests. The magnitude of the UTS obtained by the direct tensile test was smaller than that obtained by the Brazilian test. To quantify the difference among the experimental tensile strengths obtained by the three-point-bending, direct tensile, and Brazilian tests, Liao et al. [14] conducted numerical simulations. Their results showed that the three-point bending strength was the largest; however, the correlation between the direct tensile and Brazilian strengths was not clear. This difference was attributed to the heterogeneity and size effects, which violated the assumptions of the Brazilian test. However, contradictory reports of tensile strengths obtained via direct and indirect testing methods are found in the literature. For example, Fuenkajorn and Klanphumeesri [15] believed that the Brazilian strength was greater than the direct tensile strength, whereas Coviello et al. [16] held the opposite view. Andreev [6] claimed that the magnitudes of these two strengths are governed by the rock material. Even for the same rock material, the experimental tensile strength varies with different testing standards [11].

The ring tensile testing method is the other indirect method to obtain the tension strength of rocks. It was first developed by Berenbaum and Brodie [17] and Jaeger and Hoskins [18] who presented the method with complete theoretical and experimental analyses. Chen and Hsu [19] investigated the indirect tension strength of marble via a Brazilian ring disc with a hole. The experimental tensile strength varied in the ring test and was correlated with the physical parameters of limestone. Aliha et al. [20] developed a novel seesaw device for a direct ring-tension test and proposed a mathematical formulation to compute the tensile strength of the ring specimen. The experimental results were identical to the Brazilian and compression ring test results.

Four-point bending and three-point tests are two practical experimental approaches to determine the tension strength of brittle material. Four-point bending tests of marble beams were conducted by Cardani and Meda [21]. The tensile strength was found to be correlated with the dimensions of the specimens. The three-point bending strength of granite was compared with the UTS obtained from double-edge-notched specimens [22]. The former was affected by specimen size and larger than the latter. The ratio of the four-point bending test strength to the Brazilian test strength was approximately 3.81–4.26 for rectangular granite and 3.14–3.48 for cylindrical granite [23]. The experimental studies of Coviello et al. [16] demonstrated that bending tests were more reasonable in determination of the tension strength of rocks. Efe et al. [24] found that the bending strength was greater than the Brazilian test strength and UTS. In fact, the tensile strength obtained using the indirect method was greater than the UTS. Correction coefficients were suggested to convert indirect tensile strength into direct strength.

The dimension of the specimen used for the tensile test is highly dependent on the testing method. The preparation and quality of the specimen are known to be critical in experimental studies. A group of flattened sandstone cylinders was fabricated in the study of Rao et al. [25]. The uniformly distributed cross-sectional stress in the middle of the cylinder is beneficial for generating the actual tensile behavior of the rock under test. A novel double-arrow specimen was developed by Resan et al. [26] assuming a strut–tie model. The experimental tensile strength was higher than Brazilian strength but lower than that derived by the flexural test. The most reliable testing method for obtaining the UTS of rock materials is considered to be the direct tensile test. Fuenkajorn and Klanphumeesri [15] developed a delicate experimental device for the direct tensile testing of dumbbell-shaped sandstone specimens. The experimental UTS was lower than the strengths obtained by the Brazilian and ring tensile tests. Unlu and Yilmaz [27] developed a portable push–pull testing facility in which the end-fixing of the specimen was not required. The experimental testing of the UTS of rock materials is time-consuming and depends on the testing facility quality and laboratory assistant expertise. To obtain an approximate prediction of the UTS, an empirical expression based on the fundamental properties of stone is required. The Brazilian tests conducted by Palchik and Hatzor [28] demonstrated that the porosity of sandstone has a negative effect on its strength. A power function of porosity was employed to regress the Brazilian test results for rocks [29]. Using artificial neural networks, Gurocak et al. [30] conducted a statistical analysis to estimate the tensile strength of 686 rock samples.

Sandstone has been widely used in buildings as a load-bearing component. However, its tensile behavior is not adequately understood. The experimental UTS–UCS ratio was also underestimated by the empirical expressions reported in the literature. In the current study, uniaxial tensile, four-point bending, and compressive tests were conducted to characterize the UTS of sandstone. The experimental stress–strain diagrams were analyzed and compared with those published in the literature. Representative diagrams were constructed using regression analysis. Direct and indirect UTSs were obtained, and the empirical correlations with the UCS were determined on the basis of the experimental results. The current work can guide the structural design of sandstone components in buildings and other infrastructural systems.

## 2. Specimen and Laboratory Tests

### 2.1. Uniaxial Compressive Test

**Figure 1:**
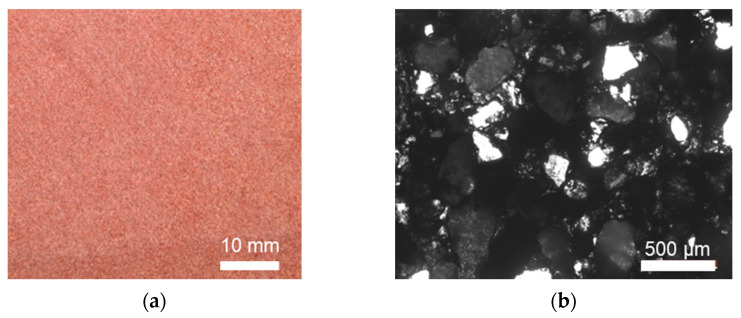
Red sandstone [40]. (**a**) Surface image. (**b**) Microscopic image.

**Figure 2:**
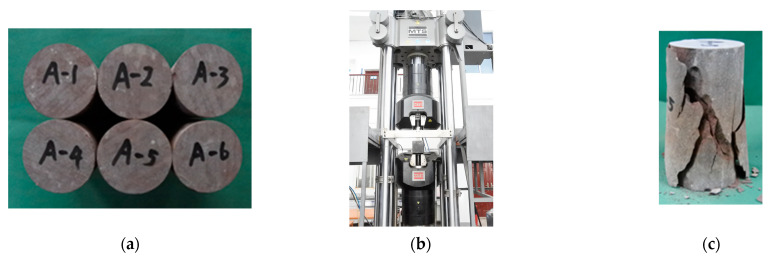
Compressive test and damage. (**a**) Sandstone cylinder [40]. (**b**) Fatigue machine. (**c**) Fracture failure.

Red sandstone, quarried from Zigong, China, has prominent mechanical and physical properties and aesthetic effect. Its mineral contents are primarily quartz, plagioclase, and calcite with percentages of 41%, 33%, and 10%, respectively. Its surface and microscopic images are shown in Figure 1. The porosity and density (2.35% and 2.40 g/cm^3^, respectively) of the red sandstone differ from those of the sandstone quarried from other places [31,32]. The specimens used for the compression, bending, and tensile tests were cut from the same source stone. Following the Chinese standard [33], six cylinders with length = 100 mm, and diameter = 50 mm, were manufactured by a professional fabricator (Figure 2a). The cylinders were carefully ground to ensure that the dimension tolerance was in agreement with the code provisions of ASTM [34] and that specified by Fairhurst and Hudson [35].

The performance of the loading machine has been a critical concern in the uniaxial compression testing of brittle materials. The post-peak branch of the stress–strain diagram cannot be facilely captured without the use sophisticated control technologies; moreover, the experimenter must possess adequate experimental skills [36,37,38]. The MTS-793 fatigue machine (Figure 2b) was used in the compressive tests of the sandstone. The maximum loading capacity was 2500 kN in two control modes (force and displacement controls). To measure the complete loading diagram, the displacement control mode was configured such that the fracture progressed until the complete failure of the cylinder was fully observed. Hairline cracks initiated at the two ends of the cylinder when 90% of the peak load was reached. Subsequently, the cracks propagated toward the middle of the cylinder with increasing load. As shown in Figure 2c, brittle fracture, which is considered common in rock materials, is the typical failure mode of a test cylinder [39].

### 2.2. Four-Point Bending Test

**Figure 3:**
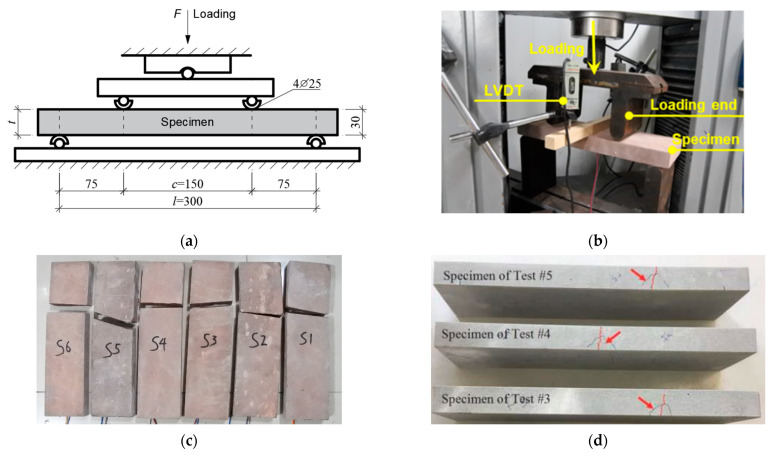
Four-point bending test. (**a**) Schematic of four-point bending test (Unit: mm). (**b**) Test overview. (**c**) Fracture failure. (**d**) Flexural bending failure of gray sandstone [44].

In the provisions of current codes, such as ASTM [41,42], the indirect tension strength obtained by the flexural bending test is actually the byproduct of experimental results [24]. The four-point bending test was performed according to ASTM [41] (Figure 3a). Five sandstone specimens (350 × 100 × 30 mm) were fabricated by skilled technicians. As summarized in Table 1, the geometry of the specimen differs from those reported in the literature. Preliminary tests showed that the rupture of the specimen occurred on the opposite side of the loading points. Accordingly, two strain gauges were fixed at two locations. The layout of the test setup is shown in Figure 3b. To measure the deflection of the specimen, two LVDTs (linear voltage differential transducers) were placed on two sides of the specimen. The loading rate was determined as 0.2 mm/min with a corresponding stress increase rate of 70 kPa/s. This loading rate is in agreement with those specified in ASTM [41] and reported by Efe et al. [24]. During the loading process, no visible deflection was observed in the middle of the specimen. Similar to compressive fracture failure, complete rupture immediately occurred after a visible crack appeared at the bottom surface of the specimen. The location of the fracture was at the opposite side of the loading ends (Figure 3c). This fracture position is identical to that observed in the three-point bending test of granite specimens [23,43] and the four-point bending test of sandstone [44].

### 2.3. Uniaxial Tension Test

In the direct tensile test, three geometries of rock specimens are generally observed: dumbbell [10], dog bone [48], and cylinder [49]. The quality and reasonableness of the geometry determine the reliability of the experimental UTS. The local unexpected stress concentrations occurring at the connections between the specimen and fixture are the main problems affecting the test results and must be avoided using an optimized geometry and reliable gripping fixture. Hoek [50] developed a dumbbell-shaped rock specimen for uniaxial tensile testing. A tapered transition from the gripping section to the test section was designed to reduce the stress concentration. The suggested length–diameter ratio was 2.0–3.0 [51]. Efe et al. [24] conducted numerical and experimental studies on rock specimens with three dumbbell geometries. They found that fracture occurred at the cross-section with the change in diameter or with the smallest diameter. The use of a cylindrical samples where tension force was applied at the ends of the specimen using epoxy is recommended by ISRM [12] and ASTM [49]. The geometrical details of the specimen for the direct tension test of the brittle material are shown in Table 2. The table indicates that the most common geometries are dumbbell and cylinder.

**Figure 4:**
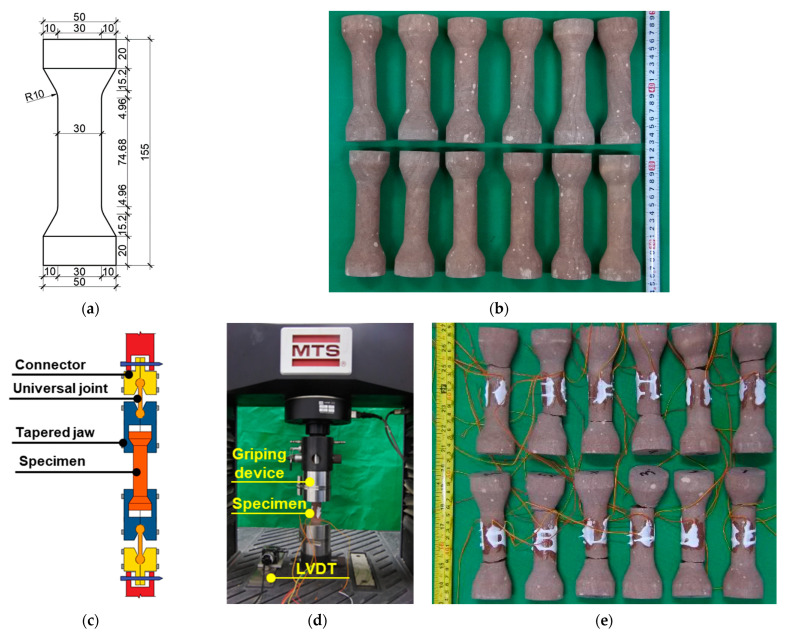
Direct tensile test. (**a**) Geometry of specimen. (**b**) Sandstone specimen. (**c**) Griping device. (**d**) Test layout. (**e**) Fracture failure of specimen.

In the direct tensile test, the geometry of the dumbbell specimen was considered as yielding the most reliable results; hence, it was used in the current study. The detailed geometry of sandstone specimens is shown in Figure 4a. The specimen was fabricated by a professional stone factory, and a water mill was used to grind the specimen and ensure that the flatness satisfied the 2020 ASTM code provisions [49] (Figure 4b). A novel gripping device was developed to grab and secure the dumbbell specimen during the tensile test (Figure 4c). The tapered jaw was calibrated, and the allowable diameter of the specimen was 25–50 mm. A universal joint can ensure that the tensile load acting on the specimen is vertical without any additional movement. The connector of the device can adapt to most universal testing machines with standard connections. MTS E45.305 was used to perform the experiments. The LVDT was fixed at both ends of the gripping device to measure the loading displacement of the facility. The actual fracture location along the specimen was unpredictable. Consequently, the strain gauges were fixed to the full length of the uniform neck of the dumbbell. A crucial factor influencing the mechanical properties of rock materials is loading rate [54,55,66]. This rate is determined to be 0.05 mm/min, which is approximately 10 N/s. Similar to the direct tensile tests reported in [24,59,67], abrupt brittle fracture was the predominant failure mode. The fracture appeared at the cross-section close to the taper outside the gripping device (Figure 4e). An identical failure mode is observed in the uniaxial tensile testing of the dumbbell stone specimen reported by Efe et al. [24].

## 3. Results and Discussions

### 3.1. Experimental Diagrams

#### 3.1.1. Uniaxial Compression Diagram

**Figure 5:**
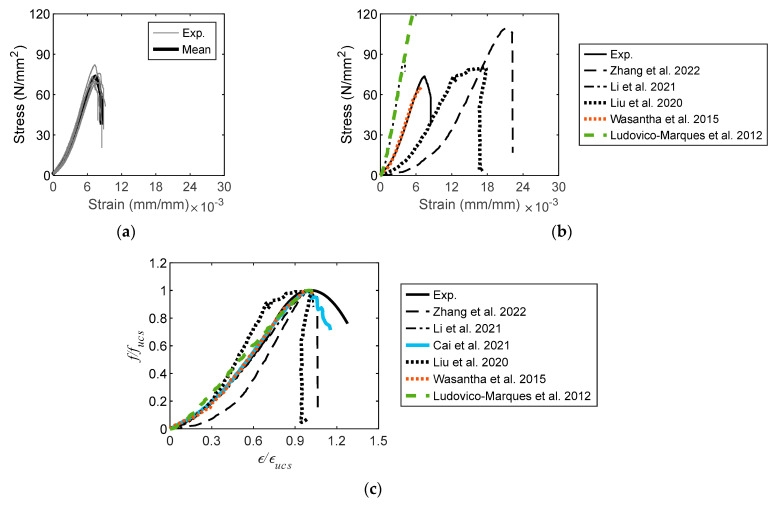
Uniaxial compression diagram [40]. (**a**) Stress–strain diagram. (**b**) Diagram comparison [31,68,69,70,71]. (**c**) Diagram regression [31,37,68,69,70,71].

The experimental stress–strain diagrams are shown in Figure 5a, from which the pre-peak and post-peak branches are successfully obtained. The diagrams converge, demonstrating that the experimental results are stable and reliable. The stress–strain diagrams published in the literature are shown in Figure 5b; the profiles of the experimental diagrams vary from those of the sandstones reported in the literature. The post-peak branches of the diagrams were not obtained by Li et al. [31], Wasantha et al. [68], or Ludovico-Marques et al. [69]. The complete diagrams were only obtained by the current study and Zhang et al. [70]. However, the profiles of the diagrams are completely different in terms of the peak stress and critical strain. The diagram from Liu et al. [71] did not have a distinct boundary between the pre- and post-peak branches, indicating that the experimental diagram was not ideal. The foregoing is identical to that in the current study. The experimental UCS range is 69.8–82.0 N/mm^2^, which approximates those reported by Li et al. [31], Wasantha et al. [68], and Liu et al. [71]. The variation in the UCS of this study is due to the variable mineral content and grain size of the sandstones quarried from different locations.

The evolution of the stress–strain diagram is not demonstrated by the mean diagram, as shown in Figure 5a. A representative diagram must be developed based on experimental diagrams. Accordingly, each diagram was normalized by the peak stress (*f*_ucs_) and corresponding critical strain (*ε*_ucs_). It was then regressed by two functions divided by the crack damage stress and strain (*ε*_cd_). The foregoing is given by

(1) $$\frac{f}{f_{ucs}}=\left\{ \begin{array}{cc} 1.13{\left(\varepsilon /\varepsilon_{\mathrm{ucs}}\right)}^{1.42}, & 0\leq \varepsilon \leq \varepsilon_{cd}\\ -3.57{\left(\varepsilon /\varepsilon_{\mathrm{ucs}}\right)}^{2}+6.92(\varepsilon /\varepsilon_{\mathrm{ucs}})-2.39, & \varepsilon >\varepsilon_{cd} \end{array}\right.$$

where *f* is the compressive stress, and *ε* denotes the compressive strain. The *R*^2^ errors of the regressed diagrams are 0.965 and 0.433 for the first and second segments, respectively. The experimental stress–strain diagrams of sandstone reported in the literature were processed using this method, as shown in Figure 5c, where the pre-peak and post-peak behaviors of the experimental diagrams are more clearly demonstrated than those in Figure 5b. The post-peak portions of the diagrams in the current study and those reported by Song et al. [72] were well measured. An abrupt dropdown was observed in the post-peak portion of other diagrams [31,68,69,70,71]. The pre-peak portions of the diagrams in the current study are identical to those reported by Li et al. [31], Cai et al. [37], and Wasantha et al. [68], demonstrating the identical loading behavior of the sandstone.

#### 3.1.2. Flexural Tension Diagram

**Figure 6:**
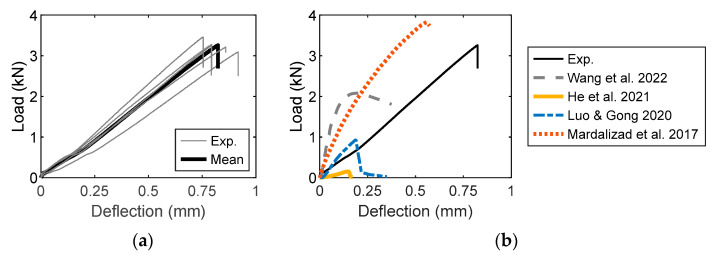

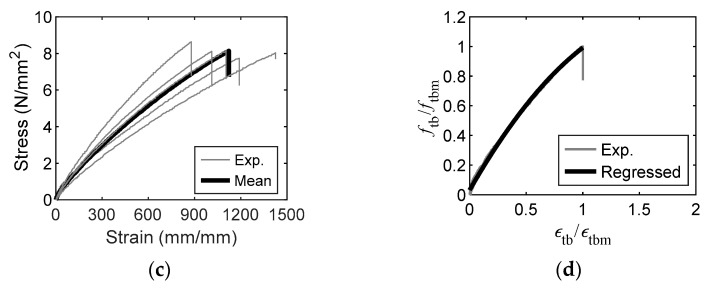
Flexural tension diagram. (**a**) Load–displacement diagram. (**b**) Diagram comparison [44,58,73,74]. (**c**) Stress–strain diagram. (**d**) Diagram regression.

The flexural tensile test is an indirect experimental method employed to investigate the tensile strength of rocks, including sandstone. The load–deflection diagram is shown in Figure 6a, where the peak load range is 2.8–3.3 kN with a corresponding deflection of 0.7–0.8 mm. The mean load–deflection was compared with the experimental results in the literature (Figure 6b). Five four-point bending tests for sandstone were conducted by Mardalizad et al. [44]. Although the peak load they obtained was relatively similar to that of the current study, the mean diagram differed considerably. For the four-point bending test with a notch in the middle [73], the experimental peak load was approximately 50% of that used in this study. The UCS of sandstone in Wang et al. [73] is 37.53 N/mm^2^; this value is approximately 50% of the UCS in the current study. The peak loads were low in the three-point bending tests for sandstone [58,74]; the existence of the middle notch actually reduced the fracture load. The change in the mineral content of sandstone may have resulted in load–deflection diagram variations.

The experimental stress–strain diagram is shown in Figure 6c. An approach identical to the preparation of a uniaxial compression diagram was employed to obtain a representative diagram of the four-point bending test. The peak stress and corresponding strain were normalized for diagram regression. The resulting diagram is shown in Figure 6d. The error of the parabolic expression used in the regression model (Equation (2)) was *R*^2^ = 0.999. The normalized diagrams for the specimens are found to be virtually identical, demonstrating that the experimental results are reliable:

(2) $$\frac{f_{tb}}{f_{tbm}}=-0.374{\left(\varepsilon_{tb}/\varepsilon_{tbm}\right)}^{2}+1.334(\varepsilon_{tb}/\varepsilon_{tbm})+0.031$$

where *f*_tb_ denotes the flexural tensile stress; *ε*_tb_ denotes the bending strain; *f*_tbm_ denotes the maximum flexural stress; and *ε*_tbm_ denotes the corresponding strain.

#### 3.1.3. Uniaxial Tension Diagram

**Figure 7:**
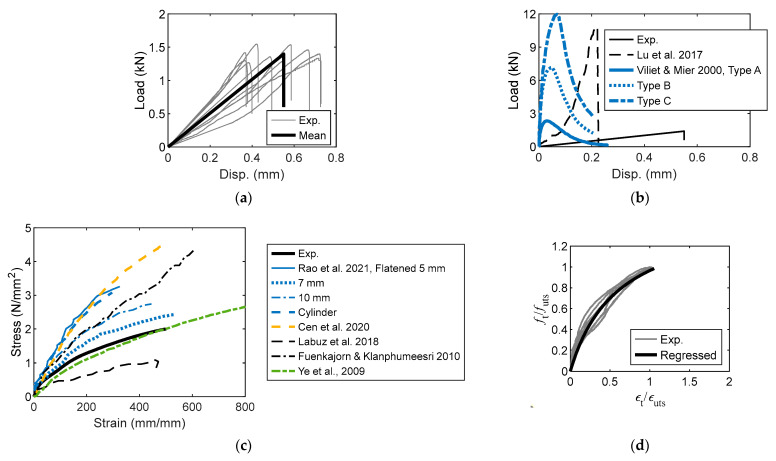
Direct tension diagram. (**a**) Load–displacement diagram. (**b**) Loading diagram comparison [63,76]. (**c**) Tensile stress–strain diagram [15,25,55,77,78]. (**d**) Diagram regression.

The load–displacement diagram is shown in Figure 7a, where the peak load range is 1.3–1.5 kN. The corresponding critical displacement range is 0.3–0.7 mm. In most diagrams, the pre-peak segment remains virtually linear up to the peak load. However, some nonlinear pre-peak portions appeared in several dumbbell specimens, indicating their progressive failure [75]. As shown in Figure 7b, the nonlinear increment of the diagram is more apparent in the Brazilian test with a larger peak load but smaller critical displacement. This is because the geometry of the sandstone specimen is different from that of the sample used in this study. In addition, the sandstone used in Lü et al. [76] was quarried in Linyi, Shandong, China; it has a density = 2.41 g/cm^3^ and different mineral contents. The dog-bone-shaped sandstone reported by van Vliet and van Mier [63] had different dimensions (Table 3). The nonlinear behavior appeared in the pre- and post-peak portions of the loading diagram. A larger peak load was obtained for specimens of small sizes. Additionally, all the experimental peak loads in the three types of specimens were larger than those in the dumbbell specimens in this study.

The stress–strain diagrams of this study and those reported in the literature are shown in Figure 7c. In all the experiments, a nonlinear tensile behavior was exhibited by the flattened and full cylinders [25,55,77], dumbbell [15], and Brazilian discs [78]. The evolution of tensile stress differs from that of compressive stress because in most circumstances, cracking initiation is an indication of tensile failure. The experimental stresses of the full and flattened sandstone cylinders are larger than the stress obtained in this study [25]. Only the diagram reported by Ye et al. [78] is identical to that in this study. However, the peak stress in the diagram of the former is 3.4 N/mm^2^, which is larger than the peak stress obtained in this study. The geometries of the diagrams of the sandstone cylinder in Ye et al. [78] and Rao et al. [25] are identical, but the peak stresses differ. The diagram of the curved sandstone dumbbell in Fuenkajorn and Klanphumeesri [15] is compared with that in this study. To obtain a representative stress–strain diagram from the uniaxial tensile test (Figure 7d), a power function model was used in the regression analysis. The *R*^2^ error is 0.942. The resulting mathematical expression is given by Equation (3):

(3) $$\frac{f_{t}}{f_{uts}}=1.533(\varepsilon_{t}/\varepsilon_{uts})/(0.608+\varepsilon_{t}/\varepsilon_{uts})$$

where *f*_t_ is the uniaxial tensile stress; *ε*_t_ is the uniaxial tensile strain; *f*_uts_ is the maximum tensile stress; and *ε*_uts_ is the corresponding strain.

### 3.2. Experimental Strength

**Figure 8:**
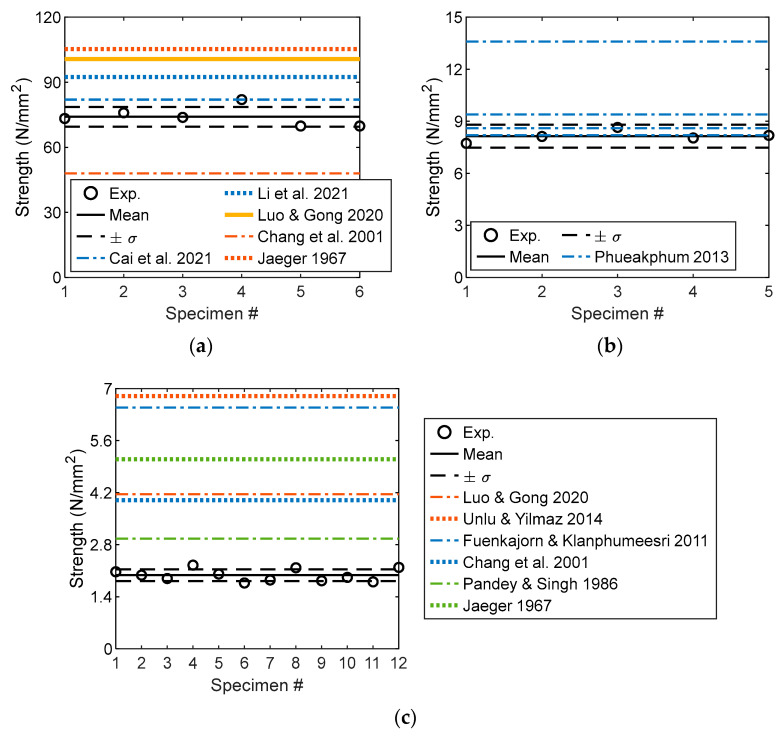
Experimental strength (Note: *σ* denotes standard deviation). (**a**) UCS [18,31,37,74,79]; (**b**) FBS [45]; (**c**) UTS [15,18,27,74,79,81].

The experimental UCS is shown in Figure 8a, where the mean UCS is 74.1 N/mm^2^ with a standard deviation of 4.5 N/mm^2^. The UCS of the sandstone reported by Cai et al. [37] approaches that found in this study. The UCS of sandstone in Chang et al. [79] is the lowest. The experimental UCS values in Li et al. [31], Luo and Gong [74], and Jaeger [18] are all greater than that of this study. In accordance with ASTM [42,80], the flexural bending strengths from the four-point and three-point bending tests are given by Equation (4):

(4) $$\sigma_{t4}=\frac{3Fl}{4bh^{2}}$$

The flexural bending strength (FBS) obtained using Equation (4) is shown in Figure 8b; the FBS range is 7.7–8.6 N/mm^2^. This FBS is identical to the experimental bending strengths of the Saraburi marble (8.2 N/mm^2^) and Phra Whihan sandstone (8.6 N/mm^2^) reported in Phueakphum et al. [45]. However, it is lower than the strengths of Phu Phan sandstone (13.6 N/mm^2^) and Phu Kradung siltstone (9.4 N/mm^2^). The experimental UTS of the sandstone is shown in Figure 8c; the mean value is 1.98 N/mm^2^ with a standard deviation of 0.16 N/mm^2^. Apparently, the magnitude of the strength found in this study is lower than those reported in the literature [81].

### 3.3. Correlations between UTS, UCS, and FBS

#### 3.3.1. UTS and UCS

The direct tensile test is known to be the most reliable approach for obtaining the UTS of rock materials. However, laboratory tests are generally expensive and time-consuming. Empirical correlations with acceptable accuracy are required to estimate the UTS of rocks without complex experimental and analytical work. Sheorey [82] investigated the strength ratio of the UCS to the UTS based on experimental sandstone data. The results demonstrated that the magnitude of the strength ratio varies in the range 2.7–39 with an average value of 14.7. However, the strength ratio was determined to be 10–50 by Vutukuri et al. [83]. The variation in the strength ratio was found to be correlated with rock type [84]. Hoek and Brown [85] established a failure criterion for intact rock. This criterion is given by

(5) $$f_{1}=f_{3}+f_{ucs}{\left(m_{i}\frac{f_{3}}{f_{ucs}}+1\right)}^{0.5}$$

where *f*_1_ and *f*_3_ denote the major and minor principal stresses, respectively, and *m*_i_ is the material constant. When *f*_1_ = 0 and *f*_3_ = *f*_uts_, Equation (5) transforms to

(6) $$m_{i}=\left({\left(\frac{f_{uts}}{f_{ucs}}\right)}^{2}-1\right)\frac{f_{ucs}}{f_{uts}}$$

For rock materials, the ratio of *f*_uts_/*f*_ucs_ is negligible; accordingly, *m*_i_ ≈ *f*_ucs_/*f*_uts_. The error is less than 1.6% when *f*_ucs_/*f*_uts_ > 8.0. For sandstone, *m*_i_ = 17 ± 4 is the recommended value [86]. Analytical studies by Cai [86] found that the strength ratio can be represented by the crack initiation stress, *f*_ci_; specifically, *f*_uts_ = *f*_ci_/8.

The fracture failure of rocks is governed by the initial appearance of microcracks [87]. According to this theory, the UTS is

(7) $$\sigma_{t}=\sqrt{\lambda E^{\prime }\gamma /c}$$

where *λ* = 2/*π*; *c* is the half-crack length; *γ* is the specific surface energy; *ν* is Poisson’s ratio; *E′* = *E* is a plane-stress problem; and *E′* = *E*/(1 − *ν*^2^) is a plane-strain problem. However, Equation (7) is not practicable because parameter c is difficult to determine in advance. Griffith’s theory in Equation (7) is extended with the assumption that the elliptical cracks propagate from the points where the maximum tensile stress is concentrated, as given by [88]:

(8) $$\begin{array}{cc} {\left(f_{1}-f_{3}\right)}^{2}-8f_{uts}\left(f_{1}+f_{3}\right)=0 & \;\mathrm{if}\;f_{1}+3f_{3}>0\\ f_{3}+f_{uts}=0 & \;\mathrm{if}\;f_{1}+3f_{3}<0 \end{array}$$

Under uniaxial and biaxial compression, *f*_3_ = 0, *f*_1_ = *f*_ucs_, and the magnitude of the ratio of *f*_ucs_/*f*_uts_ = 8.0. The three-dimensional expression in Equation (7) is developed by Murrell [89] as follows:

(9) $${\left(f_{1}-f_{2}\right)}^{2}+{\left(f_{2}-f_{3}\right)}^{2}+{\left(f_{3}-f_{2}\right)}^{2}-24f_{uts}\left(f_{1}+f_{2}+f_{3}\right)=0$$

Using Equation (9) for the uniaxial tension scenario, *f*_ucs_/*f*_uts_ = 12.0. Murrell [90] modified the Griffith criterion with an expression considering biaxial and triaxial cases. It is given by

(10) $$\left(\sqrt{1+\mu^{2}}-\mu \right)\left(f_{1}-f_{3}\right)=\alpha^{\prime }f_{uts}\sqrt{1+f_{cc}/f_{uts}}+2\mu \left(f_{3}-f_{cc}\right)$$

where *α*’ = 4.0 for the biaxial case; *α*’ = 2(2 − *ν*) for penny-shaped cracks in the triaxial case; *f*_cc_ is the crack closure stress, and *µ* is the coefficient of friction. Generally, 3.0 < *α*’ < 4.0 and 0.5 < *ν* < 1.0. Suppose 0 < *f*_cc_/*f*_uts_ < 3.0; then, the predicted strength ratio is 6.0 < *f*_ucs_/*f*_uts_ < 10.0. Mahmood et al. [91] provided an empirical expression (Equation (11)) in accordance with the uniaxial compressive and tensile tests of limestone, sandstone, and gypsum. The corresponding parameters of the expression are listed in Table 4. The empirical expression is

(11) $$f_{uts}/f_{ucs}=\frac{1}{a_{1}+b_{1}f_{ucs}}+c_{1}$$

**Figure 9:**
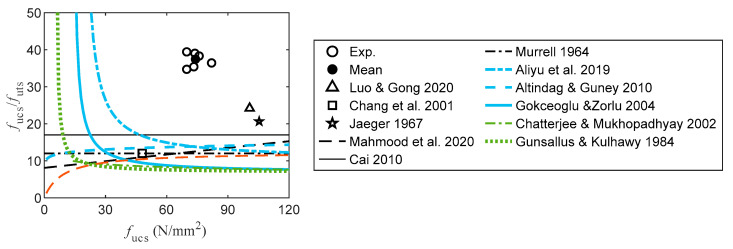
Correlation between UTS and UCS [18,74,79,86,90,91,92,93,94,95,96].

The experimental data and empirical expressions for the correlations between the UTS and UCS are shown in Figure 9. The ratios of UTS to UCS in this study all exceed the experimental results reported by Luo and Gong [74], Chang et al. [79], and Jaeger [18]. The empirical expressions of Cai [86], Murrell [90], and Mahmood et al. [91] underestimated the experimental results of the study. However, the experimental ratio in Chang et al. [79] was estimated using the expressions suggested by Murrell [90] and Mahmood et al. [91]. The variations in the physical and chemical parameters of sandstone are the main cause of the differences. To represent the correlations between the indirect UTS and UCS using the experimental data reported in the literature, the empirical power and linear expressions were regressed (Table 5). In Equations (12)–(15) and (17), the UTS is obtained using the Brazilian testing results. By contrast, the UTS in Equation (16) is derived using an empirical expression based on the Shore hardness test [92]. The experimental ratio of the UTS to the UCS is underestimated by the empirical expressions listed in Table 5. In general, the magnitude of the ratio is unreasonably large when *f*_ucs_ < 40 N/mm^2^. This magnitude is typically not a practical compression strength for medium and hard rocks, such as sandstone. Consequently, the corresponding portion of each profile is negligible.

#### 3.3.2. UTS and FBS

**Figure 10:**
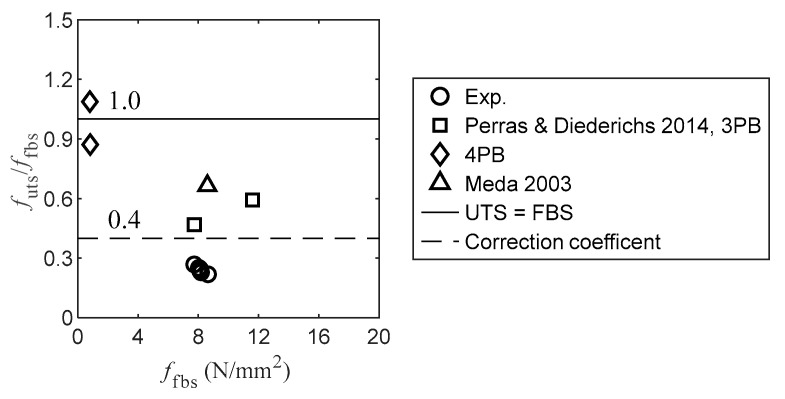
Correlation between UTS and FBS (Note: 3PB and 4PB denote three-point and four-point bending, respectively) [10,62].

The correlations between the UTS and FBS are shown in Figure 10. The indirect tension strength is generally lower than the direct tension strength [10]. The ratio of UTS to FBS varies with the rock type (Figure 10). The magnitude of the experimental ratio is approximately 0.25, which differs from the four-point and three-point bending test results. The experimental UTS and FBS of Serena sandstone in Meda [62] are shown in Figure 10, where the magnitude of the FBS is identical to that in this study. The magnitude of UTS/FBS was 0.70, which was larger than that of the current study. This is because the geometry of the specimen used for the uniaxial tensile tests is prismatic; it differs from that of the specimen used in the current study. Additionally, two types of specimens were notched in the middle. The correction coefficient, namely, the ratio of UTS to the indirect tensile strength, is provided to modify the indirect tension strength [24]. The suggested correction coefficient for the four-point bending test results is 0.4, which apparently overestimates the experimental results. Consequently, the suitable correction coefficient is 0.25, which is slightly less than the suggested correction coefficient for the three-point bending test (0.33) [24].

### 3.4. Elastic Modulus

**Figure 11:**
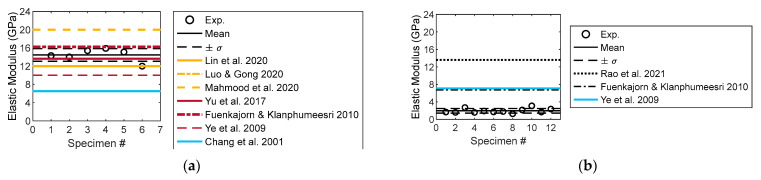
Experimental elastic moduli. (**a**) Compressive modulus [15,74,78,79,91,97,98]. (**b**) Tensile modulus [15,25,78].

Under compression and tension, the experimental elastic moduli are computed, as shown in Figure 11. The compressive elastic modulus range is 12.0–15.9 GPa with an average value of 14.45 GPa (Figure 11a). The magnitude of the modulus differs from the experimental results reported in the literature, and is generally 12–16 GPa. The experimental modulus in Chang et al. [79] was the lowest (6.5 GPa) and that in Mahmood et al. [91] was the largest (20 GPa). The foregoing indicates that the variability of the experimental modulus of rock materials is common even for the same rock type (e.g., sandstone). The computed tensile elastic modulus is as shown in Figure 11b. Similar to the tensile strength of sandstone, the magnitude of the tensile modulus is extremely small compared with the compressive modulus. The tensile modulus range is 1.59–2.36 GPa with a mean value of 1.96 GPa. Because of the experimental method and variation in the mineral content of sandstone, the experimental results are significantly less than those in the literature [15,25,78].

**Figure 12:**
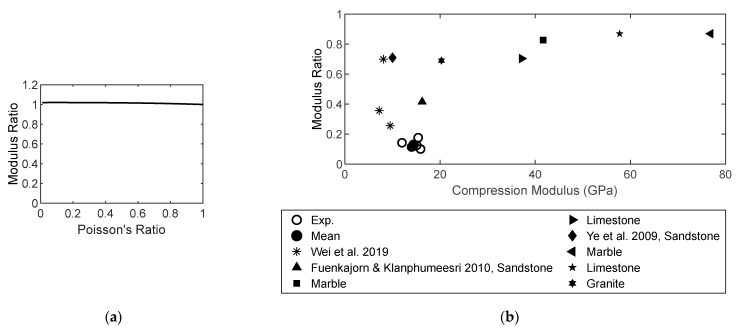
Tension–compression modular ratio. (**a**) Modular ratio and Poisson’s ratio. (**b**) Modular ratio and compressive strength [15,78,103].

Chen and Stimpson [99] conducted a numerical study to characterize the correlation between the elastic modulus in tension and compression. Asem et al. [100] computed the tensile elastic modulus using the experimental data from a four-point bending test. The modulus is given by

(18) $$E_{t}=\frac{E_{eq}{\left(1+\varepsilon_{tb}/\varepsilon_{cb}\right)}^{2}}{4(\varepsilon_{tb}/\varepsilon_{cb})}$$

where *E*_eq_ is the equivalent modulus obtained from the four-point bending test [95], and *ε*_tb_ and *ε*_cb_ are the tensile and compressive strains at the top and bottom fibers, respectively, of the specimen cross-section. When bending is symmetrical with respect to the neutral axis of the cross-section, *E*_t_ = *E*_eq_. The ratio of the tension to the compression modulus is represented by the Poisson’s ratio and crack density [100]. It is given by

(19) $$\frac{E_{t}}{E_{c}}=\left[1+\frac{16(1-\nu^{2})(1-3\nu /10)}{9(1-\nu /2)}\rho_{c}\right]$$

where *ρ*_c_ denotes the crack density of the rock material (*ρ*_c_ = 0.011 for sandstone) [101]. As suggested by [73], for sandstone, Poisson’s ratio is 0.247. However, in the study of Ye et al. [78], it was only 0.169. Generally, the range of this ratio is 0.05–0.4 for sandstone [102]. The resulting modular ratio (*E*_t_/*E*_c_) is approximately 1.0 (Figure 12a), which is apparently not sensitive to Poisson’s ratio. In Figure 12b, the experimental modular ratio of sandstone in this study is 0.12–0.14, which is smaller than that reported in the literature [15,78,103]. All the experimental modular ratios are less than 1.0 and do not agree with the computational results obtained using Equation (19). However, Yu et al. [104] used a modular ratio exceeding 1.0. In general, the magnitude of the modular ratio is observed to increase with the elastic compressive modulus. The elastic modulus of the rock material is sensitive to experimental methods [105]. Owing to the variability in the mineral contents of sandstone, an expression representing the correlation between *E*_t_/*E*_c_ and *E*_c_ is difficult to develop.

## 4. Conclusions

Red sandstone has been widely used in various infrastructural systems, such as building structures. However, the UTS is not adequately understood. Uniaxial compressive, tensile, and four-point bending tests were carried out to characterize the UTS of the red sandstone. Some conclusions are as follows:

1. The standard dimensions of the specimens for the compression and four-point bending tests were obtained from the code provisions. These provisions were applied to the fabrication of sandstone cylinders and rectangular slabs. However, the dimensions of the dumbbell specimen for the uniaxial tensile test have not been specified in the current codes.
2. The experimental stress–strain diagrams of the three types of tests were not identical to those reported in the literature. To characterize the evolution of the stress–strain profiles obtained from the uniaxial compressive, tensile, and four-point bending tests, representative expressions were developed in terms of normalized strain and strength.
3. The mean experimental UCS, UTS, and FBS were 74.1, 8.1, and 1.98 N/mm^2^, respectively. The magnitude of the UTS was greater than that of the FBS, indicating that the UTS cannot be represented by the FBS.
4. The correlations between the UTS, UCS, and FBS were analytically investigated based on the experimental results. The experimental UTS–UCS ratio (33–41) was underestimated by the empirical expressions reported in the literature. By contrast, the UTS–FBS ratio was overestimated by Efe et al. [24]. The suggested correction coefficient was 0.25 instead of 0.4.
5. The elastic tensile and compressive moduli were computed using the experimental stress–strain diagrams. The compressive modulus was generally in agreement with the experimental results reported in the literature, whereas the tensile modulus was overestimated. The experimental modular ratio, *E*_t_/*E*_c_, ranges from 0.12 to 0.14. It was not sensitive to Poisson’s ratio; however, it slightly increased with the compressive modulus.

The variation in the UCS, UTS, and FBS of sandstone is due to the variable mineral contents and grain sizes of the sandstone quarried from different locations. Accordingly, experimental test is the most reliable approach for obtaining the tensile strength of a brittle rock material. The experimental results of this study are beneficial to other types of rock materials in understanding the tensile strength.

## Figures and Tables

**Table 1:** Geometrical details of four-point bending test specimen.

Reference	*l* (mm)	*b* (mm)	*c* (mm)	*t* (mm)
Current study	300	100	150	30
Efe et al. [24]	320	102	160	32
	125	50	41.6	25
	250	50	83.3	50
Mardalizad et al. [44]	318	102	159	32
Phueakphum et al. [45]	240	150	80	20
Efimov [46]	100	20	40	20
Coviello et al. [16]	220	50	130	50
	220	50	100	50
	220	60	70	50
	160	50	70	40
Cardani and Meda [47]	1200	24	600	20
	600	24	300	40
	300	24	150	80
	150	24	75	160

*b*: stone specimen width.

**Table 2:** Geometrical details of specimens for direct tensile test.

Reference	Geometry	*D* (mm)	*L* (mm)	*L/D*	Neck Shape	Fixture
Current study	Dumbbell	30	75	2.5	Uniform neck with tapered fillet	Tapered jaw
Zhao et al. [48]	Dog bone	5 (width)	5	1.0	Uniform neck with tapered fillet	Epoxy
Wang et al. [52]	Dumbbell	40	15	0.375	Variable neck	Metal holder
Jiang et al. [53], Liu et al. [54], Cen et al. [55], and Yuan and Shi [56]	Cylinder	50	100	2	Uniform	Epoxy
Efe et al. [24]	Dumbbell	52	126	2.4	Uniform neck with tapered fillet	Jaw and loading plate
		42	90	2.1	Uniform neck with two tapered fillets	
		42	90	2.1	Variable neck with short and tapered fillet	
Rao et al. [25]	Flattened cylinder	30–44 (width)	20	—	Flat	Epoxy
Huang et al. [57]	Cylinder with two notches	50	125	2.5	Uniform	Epoxy
He et al. [58]	Dog bone	5 (width)	10	2.0	Uniform	Tapered jaw
Lan et al. [59]	Dumbbell	30	82	2.7	Variable neck	Epoxy
Cacciari and Futai [60]	Cylinder	49.4–49.6	100–115	2.0	Uniform	Epoxy
Unlu and Yilmaz [27]	Cylinder	54	120	2.2	Uniform	Epoxy
Erarslan and Williams [61]	Cylinder	52	135	2.6	Uniform	Epoxy
Fuenkajorn and Klanphumeesri [15]	Dumbbell	30	140	4.7	Variable neck	Flat bearing plate
Coviello et al. [16]	Cylinder	25, 50, 60	50, 100, 120	2.0	Uniform	Epoxy
Meda [62]	Notched slab	25.5, 51.0, 102 (width)	60, 120, 240	2.0	Notches on two sides	Epoxy
van Vliet and van Mier [63]	Dumbbell	0.6L (width)	50–1600 with two increments	1.7	Variable neck	Epoxy
Okubo and Fukui [64]	Cylinder	25	50	2.0	Uniform	Epoxy
Carpinteri and Ferro [65]	Dog bone	5, 10, 20, 40	10, 20, 40, 80	2.0	Variable neck	Epoxy
Hoek [50]	Dumbbell	22	43	2.0	Uniform neck with tapered fillet	Wedge-type grip

Note: *D* is the least or uniform diameter of the specimen, and *L* is the length of the specimen with a uniform diameter or distance between the two ends with a continuously variable section.

**Table 3:** Dog-bone-shaped sandstone specimen [63].

Type	A	B	C
Length (mm)	50	100	200
Width (mm)	30	60	120
Thickness (mm)	100	100	100

**Table 4:** Parameters of empirical expression [91].

Rock Type	*a*_1_	*b*_1_	*c*_1_	*RMSE*	*R* ^2^	*#* Data
Gypsum	63.35	0.00	0.022	0.005	0.99	85
Limestone	2.15	0.10	0.40	0.02	0.91	96
Sandstone	8.1	0.06	0.00	0.007	0.90	22

**Table 5:** Empirical expressions on UTS and UCS.

Equation No.	Expression	No. of Data Points	R^2^	Rock Type	Country of Origin	References
(12)	*f_ucs_ =* 12.4 *f_uts_* − 9	10	0.76	Sedimentary	USA	Gunsallus and Kulhawy [93]
(13)	*f_ucs_ =* 10.33 *f_uts_*^0:89^	22	0.94	Sedimentary	India	Chatterjee and Mukhopadhyay [94]
(14)	*f_ucs_ =* 6.89 *f_uts_* + 5.39	22	0.93			
(15)	*f_ucs_ =* 6.8 *f_uts_* + 13.5	82	0.65	Sedimentary (Greywacke)	Turkey	Gokceoglu and Zorlu [95]
(16)	*f_ucs_ =* 12.308 *f_uts_*^1.0725^	143	0.90	Mixed	Several countries	Altindag and Guney [92]
(17)	*f_ucs_ =* 10.4 *f_uts_* + 18.2	7	0.63	Sedimentary	UK, France, and Denmark	Aliyu et al. [96]

Note: *Mixed* denotes that several rock types, such as igneous, sedimentary, and metamorphic rocks, are considered.

## Data Availability

Data will be available on request to the corresponding authors.
